# Barriers and facilitators to hepatitis C screening and treatment for people with lived experience of homelessness: A mixed‐methods systematic review

**DOI:** 10.1111/hex.13400

**Published:** 2021-12-03

**Authors:** Martha Paisi, Neeltje Crombag, Lorna Burns, Annick Bogaerts, Lyndsey Withers, Laura Bates, Daniel Crowley, Robert Witton, Jill Shawe

**Affiliations:** ^1^ School of Nursing and Midwifery University of Plymouth Plymouth UK; ^2^ Peninsula Dental School University of Plymouth Plymouth UK; ^3^ Department of Development and Regeneration, Urogenital, Abdominal and Plastic Surgery KU Leuven Leuven Belgium; ^4^ Department of Development and Regeneration, Unit Woman and Child KU Leuven Leuven Belgium; ^5^ Faculty of Medicine and Health Sciences, Centre for Research and Innovation in Care (CRIC) University of Antwerp Antwerp Belgium; ^6^ Community volunteer/research partner Plymouth UK; ^7^ Hepatology Nursing Team University Hospitals Plymouth NHS Trust Plymouth UK; ^8^ The Hepatitis C Trust Plymouth UK; ^9^ Royal Cornwall NHS Trust Cornwall UK

**Keywords:** adult, delivery of health care, hepatitis C, homeless persons, humans

## Abstract

**Background:**

People experiencing homelessness have an increased risk of hepatitis C virus (HCV) infection, with rates higher than the general population. However, their access to HCV diagnosis is limited and treatment uptake is low.

**Objectives:**

To identify and describe the barriers and facilitators for HCV screening and treatment for adults with lived experience of homelessness in highly developed countries.

**Methods:**

Bibliographic databases (Embase, MEDLINE, CINAHL and SocINDEX) and grey literature (Google, EThOS, the Health Foundation, Social Care Online, the World Health Organisation, Shelter, Crisis and Pathway) were searched. Two reviewers independently screened and appraised all studies. The Critical Appraisal Skills Programme tool and the Joanna Briggs Institute checklist were used. The analysis involved a three‐stage process: coding, theme generation and theme mapping under Penchansky and Thomas's modified access model.

**Results:**

Twelve papers/reports were included in the review. Several interacting factors influence access of people with lived experience of homelessness to HCV testing and treatment. Some mirror those identified for the general population. The precarious conditions associated with the lived experience of homelessness along with the rigidity of hospital settings and lack of awareness emerged as dominant barriers. Flexibility, outreach, effective communication, tailoring and integration of services were found to be important facilitators. Evidence from Black, Asian and minority ethnic groups is limited.

**Conclusions:**

People experiencing homelessness face multiple barriers in accessing and completing HCV treatment, relating to both their lived experience and characteristics of health systems. Although some barriers are readily amenable to change, others are more difficult to modify. The facilitators identified could inform future targeted measures to improve HCV diagnosis and treatment for people experiencing homelessness. Research is warranted into successful models to promote screening, diagnosis and treatment.

**Patient or Public Contribution:**

Our team includes a peer advocate, a hepatology nurse and a community volunteer, all with significant experience in promoting and engaging in HCV care and outreach for people experiencing homelessness. They contributed to the protocol, interpretation and reporting of the review findings.

## 1 INTRODUCTION

Hepatitis C virus (HCV) is a blood‐borne virus that can cause liver disease.^1^ Intravenous drug use is the main mode of transmission.^2^ In 2015, it was estimated that 71 million people worldwide had a chronic HCV infection.^3^ This is associated with a significant risk of developing liver cirrhosis and cancer.^4^ People with a history of homelessness in high‐income countries experience severe inequities across a number of health conditions, including HCV.^5^ This is due to various interacting socioeconomic factors that shape conditions of daily life, many being beyond an individual's control.^6^, ^7^

Intravenous drug use is highly prevalent among the homeless community, increasing their risk of infection with blood‐borne viruses.^8^, ^9^, ^10^ Although the overlap between the number of people experiencing homelessness and those using drugs is difficult to estimate, the UK and international literature indicate that it is significant.^11^ A systematic review and meta‐analysis conducted in 2012, found that the prevalence of HCV in homeless populations ranged from 3.9% to 36.2%.^12^ A more recent review in the United States showed similarly wide‐ranging figures from 9.8% to 52.5%.^13^ In homeless shelters in London, 13% of those who agreed to be screened (*N* = 491) had past or current HCV infection.^14^ The highest risk of HCV was identified in individuals reporting injecting drug use. However, even those without injecting drug history had higher levels of HCV than the general population (3% vs. 0.4%).^14^

A new class of drugs to treat HCV: direct‐acting antivirals (DAAs) are now available. They require a shorter treatment time, with better success rates and fewer adverse effects than interferon. However, access to HCV diagnosis and treatment among the homeless community remains low. In the study of Aldridge and colleagues,^14^ participants diagnosed with current HCV, showed poor engagement with health services, with over half of those referred to specialist services either not attending appointments or being lost in follow‐up. This concurs with other studies showing suboptimal uptake of HCV treatment among people experiencing homelessness.^15^, ^16^ Some factors influencing HCV testing, management and uptake of care have been identified at the societal level (e.g., social stigma against people experiencing homelessness).^17^, ^18^ Others have been identified at levels of the system (e.g., logistics in booking appointments, workforce constraints at homeless shelters). Individual factors (e.g., precarious living conditions, competing priorities, limited knowledge and misconceptions regarding HCV) are also evident.^17^, ^18^

Equity of access is a central objective of many healthcare systems.^19^ Access has been conceptualized in various ways, demonstrating its complexity.^20^ Most commonly, it is described as the interaction of factors that impact entry to or utilization of a health system. Some authors interpret access as the population's ability to seek and secure care, while others put more emphasis on the characteristics of the health system that influence utilization of services.^20^ Some conceptualize access as the ‘fit’ between the patients' needs and the characteristics of health systems,^21^ a notion whose complexity highlights the importance of analysing access from a multidimensional perspective.

Despite the high prevalence of HCV among people experiencing homelessness, a significant number remain undiagnosed and hence untreated, with reasons for the low engagement and uptake of HCV care being unclear.^22^ Previous systematic reviews have focused either on people who inject drugs or those leaving prison.^23^, ^24^ A better understanding of the factors influencing HCV screening, and treatment uptake and adherence among the wider at‐risk population of people experiencing homelessness, is crucial to guide the development of effective programmes tailored to their complex health needs.^17^ Universal access to affordable diagnostics and treatment is also important in achieving the WHO's 2030 target for elimination of HCV and reducing health inequities.^25^

Therefore, this systematic review aimed to answer the question:

What are the barriers and facilitators for HCV screening and treatment for adults with lived experience of homelessness in highly developed countries?

## 2 METHODS

### 2.1 Protocol and registration

The review protocol was registered a priori with PROSPERO (registration number: CRD42020221767). The conduct and reporting of the review was guided by ENTREQ^26^ and PRISMA guidelines.^27^

### 2.2 Eligibility criteria

Eligibility criteria were used at both title/abstract and full‐text levels as described in Table 1. There were no date or language restrictions.

**Table 1:** Eligibility criteria

Population/participants	Inclusion: Adults aged ≥18 years with current or previous experience of homelessness, and/or staff, volunteers and healthcare professionals working with homeless populations. Exclusion: Nonadult populations (<18 years). The European Typology of Homelessness was used, which includes the following living situations: ‘• rooflessness (without a shelter of any kind, sleeping rough) • houselessness (with a place to sleep but temporary in institutions or shelters) • living in insecure housing (threatened with severe exclusion due to insecure tenancies, eviction, domestic violence) • living in inadequate housing (in caravans on illegal campsites, in unfit housing, in extreme overcrowding)’.^28^
Phenomenon of interest	Access (to a service, provider or an institution) and utilization (realized access) of HCV screening and treatment among people with lived experience of homelessness.
Outcomes	Perceived barriers and facilitators to HCV screening and treatment for people with lived experience of homelessness from their perspective, and/or that of support workers and volunteers, and healthcare providers.
Type of study	Inclusion: Empirical studies using qualitative, quantitative and mixed methods. Exclusion: Reviews, letters, commentaries and editorials, conference abstracts.
Location of study	Inclusion: Countries of very high Human Development Index (HDI) to improve transferability of findings to advanced healthcare systems and services. Exclusion: Countries of high, medium or low HDI. HDI Table 1: Human Development Index and its components. Available at http://hdr.undp.org/en/composite/HDI

Abbreviation: HCV, hepatitis C virus.

### 2.3 Information sources

The literature searches were designed and undertaken by an information specialist (L. Burns) on 3 December 2020 (from the inception of the respective database to December 2020). The databases searched were Embase, MEDLINE, CINAHL and SocINDEX. Grey literature was searched using Google, EThOS, the Health Foundation, Social Care Online, the World Health Organisation, Shelter, Crisis and Pathway. Citation searching of included studies and relevant systematic reviews was also conducted.

### 2.4 Search

The search used both index and title abstract terms for the concepts of homelessness and HCV. Full details of the search strategies used in all databases are provided in Supporting Information File 1.

### 2.5 Study selection and data collection process

Search results were collected and deduplicated in EndNote and then uploaded onto Rayyan for screening.^29^ Screening on title/abstract and full text, was conducted by two independent reviewers (M. P. and N. C.). Any disagreements were resolved through discussion, consulting a third reviewer (J. S.) if there were no consensus. Data from studies that were included in the review were extracted using a pilot‐tested form. Information extracted included author, year, setting, type of homelessness, sampling, data collection method(s), participant characteristics, barriers and facilitators.

### 2.6 Critical appraisal

The studies were critically appraised independently (M. P. and N. C.) using the Critical Appraisal Assessing Skills Programme tool for qualitative studies and randomized controlled trials (RCTs)^30^ and the Joanna Briggs Institute checklist for cross‐sectional studies.^31^ Agreements were reached through discussion and consultation with a third reviewer (J. S.) when required. No studies were excluded through the critical appraisal.

### 2.7 Synthesis of findings

Included full‐text qualitative articles were uploaded onto NVivo 12 software (QSR International Pty Ltd, 2020). The analysis involved a three‐stage process: coding, theme generation and theme mapping.

#### 2.7.1 Coding

Firstly, participant quotations and authors' interpretations were inductively coded line‐by‐line by two independent reviewers (M. P. and N. C.)^32^ using semantic analysis.^33^ Descriptive coding labels were used to enable both the codes and future themes to be freely formed, rather than influenced by an a priori deductive framework developed by the authors. This aligned with our research aims to appropriately utilize and build upon existing knowledge published in this field, by synthesizing existing findings.

#### 2.7.2 Theme development

In the second stage of analysis, reviewers drew upon an adjusted approach to thematic synthesis^32^ to facilitate identification of patterns within a data set^34^ and enable researchers to stay close to the findings of primary studies, transparently linking them to conclusions made.^32^

Initially, M. P. and N. C. independently reviewed whether inductive codes could be grouped under the headings ‘barriers’ and ‘facilitators’, addressing the key research question of the study. They independently examined the similarities and differences between the codes grouped under these two headings to build descriptive themes, which captured patterns of findings across the studies.

The reviewers then discussed their decisions on grouping the codes and emergent themes, initially comparing their themes and addressing variances by adjusting/adding/merging their combined themes to reach a consensus on each. They subsequently reviewed and agreed whether each theme should be classified as a barrier or facilitator. Through these independent and joint processes, the reviewers kept the research question and aim of the study in mind. This approach to synthesizing the findings enabled this study's findings to move beyond the content of the original studies,^32^ providing analytic (latent)^33^ insights.

Quantitative results were inputted in an Excel file independently by M. P. and N. C., following consensus meetings to ensure consistency. These were codified (i.e., data transformation) and grouped, based on whether they were relatable to the qualitative ‘synthesised’ themes. The findings for each theme were then examined to establish whether they reinforced or challenged the findings each theme presented. Thus, the review used an integrated design whereby both quantitative and qualitative data were assimilated into a single synthesis^35^, ^36^ in which they could be ‘‘able to confirm, extend, or refute each other’’.^35^

#### 2.7.3 Theme mapping

Following the completion of the inductive process, it became apparent that the emergent themes were relatable to Penchansky and Thomas's modified access model, which interprets access as the degree of ‘fit’ between the patient and the service.^21^, ^37^ To explore the extent of the fit between this study's ‘synthesised themes’ (which were not changed to fit the model), they were mapped to each dimension included within the ‘modified access’ model: (1) awareness, effective communication with relevant users, including consideration of context and health literacy; (2) acceptability, the attitude of the patient towards the care provider and service characteristics and the attitude of the provider towards patients' personal characteristics; (3) accommodation (how well the service is organized to access patients and how well patients are able to use the services); (4) affordability, direct and indirect costs for the patients; (5) accessibility, the proximity to the patient and (6) availability, sufficient supply of services required to meet patient needs.^21^, ^37^, ^38^ Although independent, these dimensions are interrelated and each is important to assess the achievement of access.^37^ The model's core principle is to optimize access by accounting for these dimensions.^37^ Therefore, it was deemed useful to undertake the mapping and organize the findings through this established model to draw attention to issues relating to the model that had not been addressed in the existing literature and/or service provision, and identify measures that could improve access to HCV care among people experiencing homelessness. The wider team was invited to review the findings and contribute to their interpretation; results were adjusted accordingly.

## 3 RESULTS

**Figure 1:**
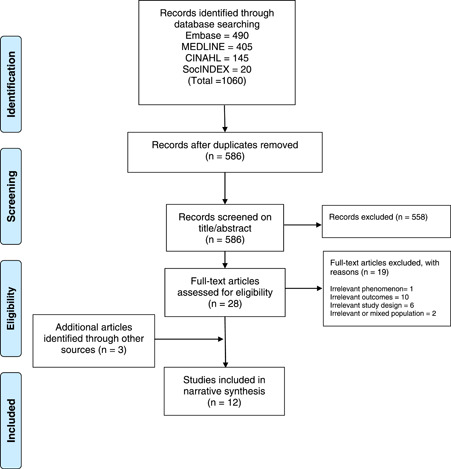
PRISMA flow chart

The search results at each stage are shown in the PRISMA figure below (Figure 1).

Initially, 1060 papers were identified. Following deduplication, 586 papers were screened on title and abstract. Screening on full‐text was conducted on 28 papers, of which 9 were included in the review, plus 3 identified through searching grey literature, giving a total of 12.

The studies used qualitative (*N* = 9) or quantitative (*N* = 3) methods; one of the latter was an RCT. The studies took place in the United States (*N* = 6), the United Kingdom (*N* = 5) and Ireland (*N* = 1) and in various settings/contexts including: clinics within a programme of healthcare for people experiencing homelessness (a freestanding outpatient clinic, a medical respite facility, a clinic located in a day shelter and a clinic within an emergency shelter) (*N* = 1); homeless shelters (*N* = 3); research cohorts (*N* = 4); outreach hepatitis C testing and intervention (for those in temporary accommodation) (*N* = 1); outreach services for drug use and homelessness offering point of care HCV, hepatitis B virus and HIV testing (*N* = 1); an outreach HCV treatment clinic established within a primary care facility (*N* = 1); a community‐based partnership offering HCV treatment and support to people who inject drugs in a drug and alcohol support programme plus a community‐based HCV outreach service (*N* = 1). Studies commonly adopted purposive sampling. In the majority, the target population was living in unstable housing. In most studies (*N* = 9), participants included those with experience of homelessness and ranged in age from 26 to 65+ years. In one study, one of the participants was in the age category of 16–25 years. Two of the studies focused on people who inject drugs, the majority of whom were experiencing or had experienced homelessness. Five studies focused on or included healthcare providers, support staff (including peer supporters) or both. The others (*N* = 7) focused exclusively on people who have experienced homelessness. In total, 986 people with experience of homelessness and 104 staff members participated in the studies. No study appeared to have included people sleeping rough. However, the participants' detailed living situation was not always reported. In eight out of the nine studies that included people with experience of homelessness and from which data could be extrapolated, the majority of participants were males (for the other one, there was an equal representation of males and females). Out of eight studies that provided data on ethnic background, the participants were predominantly White/Caucasian in five, predominantly of Black or racial/ethnic minority background in two, and in the remaining one, the distribution was almost equal. Participant and study characteristics are detailed in Supporting Information File 2.

The findings from the critical appraisal were mixed (Supporting Information File 3), with quantitative studies (except the RCT) commonly scoring lower on their quality. In qualitative studies, the relationship between the participants and researchers was commonly not reported. Insufficient reporting rendered the quality assessment of two out of the three grey literature reports difficult. All studies contributed to the review's findings; however, those of lower quality provided less data.

Table 2 illustrates the subthemes identified under each dimension of Pechanksy and Thomas' modified model. Illustrative quotes for each domain are included in Supporting Information File 4.

**Table 2:** Subthemes identified from data analysis

Dimension	Subthemes
Awareness	
Barriers	Limited knowledge regarding HCV and associated care among people experiencing homelessness and among support staff Misconceptions Negative stories about experiences of interferon treatment Limited advocacy for HCV services by shelter staff Fear of receiving positive results
Facilitators	Raising awareness among people experiencing homelessness and among shelter staff about HCV, treatment, etc. Improving awareness about addiction issues among service providers Outreach activities
Acceptability	
Barriers	Mistrust of healthcare providers and government institutions Perceived stigma and discrimination Fear of side effects of treatment Strict eligibility criteria
Facilitators	Effective communication and relationships with staff Patient‐centred services Nourishing relationships with partners and families Prompts by providers Integrating rapid HCV testing in the intake process of shelter settings Transitioning into a ‘healthier’ life
Accommodation	
Barriers	Lived experience of homelessness and associated morbidities Unstable housing Incarceration Illegal residency status Limited language skills Inflexibility with the appointment system and timings Lack of appropriate infrastructure to treat HCV and workforce constraints at the shelter level Shelter policies and rules, e.g., around drug use
Facilitators	Providers' organizational leadership and culture Multiagency partnership building Information sharing Peer support Ensuring privacy Reminders Establishing clear communication channels
Affordability	
Barriers	Perceived cost of treatment High out‐of‐pocket expenses Lack of insurance coverage Strict insurance requirements
Facilitators	Improving awareness of welfare programmes and resources Providing support with accommodation or transport Financial incentives
Accessibility	
Barriers	Distant location of clinic or hospital Lack of transport
Facilitators	Adaptable model of delivery Continuity of care Proximity of clinics Integration of services at one location
Availability	
Barriers	Intermittent attendance Long time between diagnosis and treatment Long time to wait on the day
Facilitators	Short waiting times Flexibility and adaptability

Abbreviation: HCV, hepatitis C virus.

### 3.1 Awareness (communication and information)

#### 3.1.1 Barriers

Limited knowledge and/or misconceptions regarding HCV can make people experiencing homelessness reluctant to get tested. This may relate to knowledge on modes of transmission, testing, diagnosis and availability and advances in treatment.^17^, ^18^, ^39^ Limited advocacy for services by shelter staff, who themselves reported their own low HCV treatment knowledge, may be a barrier to testing and treatment.^17^

Negative stories about past experiences of interferon treatment (i.e., long duration; adverse effects), can affect the current uptake of screening, testing and treatment.^17^ People experiencing homelessness reported that fear of a positive diagnosis and the consequences of being infected demotivated some people from getting tested, while others did not feel the need to get tested due to low perceived risk.^18^

#### 3.1.2 Facilitators

Homeless shelter users reported that education is crucial in motivating people to get tested and treated for HCV.^18^ Preventing transmission to others, along with awareness of the adverse effects of untreated HCV can significantly motivate people to be tested and treated to prevent their own disease from progressing.^18^ Raising awareness that HCV is curable has been recommended.^17^, ^18^ Increasing awareness and understanding among patients, providers and key stakeholders of the benefits of treatment with DAA agents, including duration of treatment and less challenging side effects, is important.^17^, ^18^ HCV outreach educational programmes in settings frequented by people experiencing homelessness^18^ can give the opportunity to spend time talking to people and dispelling misinformation about HCV as well as addressing stigma.^39^

The importance has been highlighted of nurses who are delivering HCV treatment also having knowledge of addiction issues, and for key workers to have a basic understanding of treatment.^40^ Providing education for shelter staff can increase their self‐efficacy to support their clients' awareness and treatment needs.^17^ Providing information in advance about HCV, what is going to take place, and the importance of getting tested, can save time on the day and encourage uptake of testing.^39^

### 3.2 Acceptability (consumer perception)

#### 3.2.1 Barriers

People experiencing homelessness report mistrust of healthcare providers and government institutions as a barrier to accessing care or seeking treatment for HCV. Some attribute this to inconsistent information from the medical community regarding the mode of transmission and available treatments.^18^, ^41^ Social services outreach workers perceived healthcare providers to have stigmatizing attitudes towards homeless people who use substances. This can deter patients from treatment uptake.^17^ Service users also stated that they experienced HCV stigma from medical providers through negative attitudes and discriminatory treatment.^40^ HCV itself was perceived by service users as a ‘stigmatised condition’ due to its link with intravenous drug use.^40^ Stigma can negatively affect individuals' decisions to disclose their HCV status, or to access care and take up treatment.^40^ Fear of side effects of treatment is another reason for not attending specialist appointments and accessing treatment.^42^

There are reports of service providers being reluctant to initiate treatment for people who are actively using drugs, due to concerns about nonadherence to treatment or risk of reinfection.^17^ ‘Stability’ was described by service providers as a key factor in deciding whether someone had the physical and mental ability to undergo what could be a strenuous treatment regime (especially in the interferon treatment era).^40^ However, service users perceived eligibility criteria such as abstinence from alcohol or drugs as unreasonable or unattainable, leading many to disengage from the service and from the hope of treatment.^40^

#### 3.2.2 Facilitators

In view of the potential impact of stigma and discrimination, providers highlighted the importance of a nonjudgmental approach and of establishing rapport with clients before commencing treatment, primarily through personal interaction.^40^ Such attitudes, perceived as positive by service users, are vital in encouraging access and uptake of HCV treatment.^40^ Developing trusting relationships between the patient, provider and healthcare system, and providing a culturally informed treatment that takes into consideration patients' backgrounds and needs, can help mitigate stigma.^18^ Service users have reported that patient‐centred services can promote the development of a caring relationship between providers and patients.^43^

Service providers reported an understanding of issues around drug use as important in establishing rapport when working with this group, and being pragmatic to achieve the best outcome in each circumstance.^40^ Familiarity with the setting and personal relationships with staff positively impact clients' perceptions of a clinic and can encourage future testing, as can positive relationships with support workers, highlighting the benefits of community‐based services.^18^, ^40^, ^44^

Those having prior experience with HCV rapid testing consider that expanding it to become a part of the intake process in shelter settings could enhance testing and linkage to care for those at the highest risk of infection.^18^ Rapid testing procedures can also encourage repeat testing in the future.^18^ For people interested in HCV treatment, motivators influencing their willingness to receive it include looking after their health, advice by a provider to get treated, and lack of side effects.^45^ Other factors motivating individuals to complete their treatment include ‘redefining the self’: getting rid of the ‘virus as part of a complete transition away from a former self towards a healthier self‐concept’, including attaining stable housing and securing employment. For this, the supportive nature of the clinical setting is critical, as are sustaining and nourishing relationships with partners and families, pursuit of ‘abstinence from substance use as a life project which mutually reinforced their desire to complete HCV treatment’, and harm reduction to avoid HCV reinfection.^43^

### 3.3 Accommodation (organization)

#### 3.3.1 Barriers

The lived experience of homelessness and associated morbidities, such as addictions, mental health issues and chronic physical health conditions have been reported as barriers to HCV testing and treatment, including attending appointments.^18^, ^41^, ^42^ Unstable housing has been reported as a common barrier to attending appointments and accessing care.^42^ The impact of homelessness on individuals' ability to engage with services is further exacerbated by competing priorities, such as securing food or managing personal hygiene,^17^ while forgetfulness can lead to nonattendance.^42^ Some service users reported that the hostel milieu can negatively impact their treatment experience and be a barrier to treatment uptake (e.g., susceptibility to resuming drug use while living in a hostel with others actively using drugs).^40^

Incarceration among this community was reported to inhibit treatment access and/or completion, or attending appointments.^40^, ^41^, ^42^ Someone with illegal residency could be deported while receiving a course of treatment.^34^, ^39^ Language barriers exacerbated by lack of or inadequate interpretation services can pose challenges for providers, especially around consent and treatment decision‐making.^40^

The precarious conditions associated with the lived experience of homelessness and the referral process exacerbate barriers to engagement with treatment.^42^ Logistics in booking hospital appointments, considered both costly (especially when using personal mobile phones) and time‐consuming, were reported as discouraging service users from making an appointment, impacting engagement with services.^40^ Appointment timings, specifically early morning ones, were reported as problematic, making attendance difficult,^40^ exacerbated by chaotic lifestyles and competing priorities. Moreover, strict appointment schedules in the hospital setting can impede this patient group, who may find it challenging to arrive for a particular, say, 15‐min slot.^40^

At the shelter level, lack of appropriate infrastructure (e.g., a system to store and dispense medications) and workforce constraints, were identified as significant barriers to expanding services in these settings.^17^ Providers reported on the challenges of inadequate time for screening, testing and treating patients with complex care needs.^17^ Furthermore, shelter policies (e.g., regarding admission) and rules (e.g., on certain behaviours, including drug use and violent behaviour) mean that the patients may not be able to complete their treatment course while being a resident.^17^, ^18^ Providers reported service users' depression and addiction, particularly to drugs and alcohol, with a presumed impact on medication adherence, as common reasons to withhold therapy.^46^

#### 3.3.2 Facilitators

Provider organizations' ‘leadership and culture’ were acknowledged as the main facilitators of onsite HCV management to optimize patient care.^17^ Multiagency working and partnership building (e.g., with social service providers and outreach workers) were reported as very important and beneficial to encouraging HCV screening, follow‐up care and medication adherence, as well as improving healthcare support to patients and signposting.^17^, ^39^, ^40^, ^44^ Information sharing within and between service providers can enhance patient care and avoid challenges caused by a lack of data‐sharing IT systems.^40^

Peer support is effective in improving engagement and helping people get into treatment,^39^ thereby increasing the probability of a successful treatment outcome.^47^ Peer advocates themselves reported that self‐disclosure of shared experiences of adversity or trauma enables them to establish rapport and equality in relationships with clients.^48^ Because peer advocates are not part of the healthcare system nor directly associated with public services, with which some people have had previous negative experience, they are able to develop an affinity with people based on common experiences.^39^ They can guide people through the care pathway, accompany them to appointments, advocate for them and keep an eye on treatment progress.^39^ Also, education provided by peers could improve HCV knowledge, enhance trust and reduce feelings of stigmatization.^17^

Ensuring privacy in the clinic setting (e.g., not specifying the medical reason for attendance) can help mitigate the fear of the stigma associated with HCV infection.^44^ Reminders directly from the clinic and through support workers, although not always common practice, were identified as key in ensuring attendance, by both service users and providers, noting clients' poor memories and chaotic lifestyles.^40^ Reminders can also encourage patients who may be experiencing side effects or other problems with their treatment to seek help.^40^ Providers reported it as useful to obtain permission and information from patients on the first meeting about ways to reach them, should they disengage from care or not be contactable through regular channels.^40^

### 3.4 Affordability (financial cost)

#### 3.4.1 Barriers

Finance‐related issues vary across countries and have been reported in some cases as barriers to accessing HCV healthcare.^18^, ^41^ If people are unaware that there are no charges for treatment, cost and ability to pay can remain a perceived barrier to seeking treatment.^18^ However, in some instances, even when there were treatment costs, participants were not aware of them or of strict eligibility criteria, so they did not act as significant barriers to seeking care.^18^ Lack of insurance coverage and high out‐of‐pocket expenses, compounded by insurance policy requirements (e.g., clean drug screens), have also been reported as important barriers to receipt of HCV care.^17^, ^40^

#### 3.4.2 Facilitators

Raising awareness, where applicable, that treatment medications are free has been recommended.^18^ Awareness of welfare programmes and resources available to pay for treatment is a key facilitator. Financial incentives (e.g., gift cards) have been highlighted as a strong motivator to promote engagement of people experiencing homelessness in HCV testing, education and follow‐up care.^17^, ^18^, ^40^ Providing support with accommodation or transport for the duration of HCV treatment, could act as an incentive to accepting treatment and a deciding factor for some in completing treatment.^40^

### 3.5 Accessibility (location)

#### 3.5.1 Barriers

The location of a clinic or hospital may make travel challenging for patients, and transport has been reported as a barrier to accessing HCV healthcare.^41^ Some patients said that they would have had difficulty attending hospital appointments due to the distance involved.^44^

#### 3.5.2 Facilitators

An adaptable model of delivery has been reported as particularly important in continuity of care, as it enables staff to deliver services in any context, for example during pandemics or with cost‐saving reductions.^39^ Both patients and providers perceived it as beneficial to treatment completion to have consistent access to healthcare teams^39^ and a designated HCV coordinator.^17^, ^40^ The latter can also address workforce constraints and facilitate comprehensive care in terms of screening, education, and so forth.^17^, ^40^ Continuity of care is perceived as very important by patients, not only because of the distress caused by sharing personal and sensitive information with multiple people, but also because they can be monitored more effectively by staff who already know them.^40^

The proximity of a clinic to patients experiencing homelessness has been identified by healthcare providers as a positive aspect of an outreach service.^44^ They also reported that it was beneficial for patients to be able to see a doctor or a nurse in the same location where they were accessing HCV treatment.^44^

### 3.6 Availability (supply and demand)

#### 3.6.1 Barriers

Erratic attendance was recognized as a significant barrier to accessing hospital‐based HCV treatment. Service providers reported a long time interval between setting up and the first treatment appointment as increasing the likelihood of a patient failing to attend, and patients reported that, without reminders, they found it difficult to remember events far in advance.^40^ Lengthy waiting times, especially for people who use drugs, were described by both service users and providers as a deterrent to attending appointments, due to unease in having to wait in an unfamiliar environment, the anxiety of the appointment itself and, potentially, physical discomfort due to withdrawal.^40^

#### 3.6.2 Facilitators

Service providers highlighted that adaptability to patients' chaotic lifestyle and flexibility regarding how appointments are organized are key to a clinic's success.^34^, ^39^ Examples include overbooking with no penalty for missing the first clinic, and having a drop‐in clinic alongside scheduled (or timed) appointments, meaning that even if patients miss their set time they are still able to see staff. Patients also perceive this as beneficial in terms of treatment continuity.^40^ Short waiting times to access treatment were also appreciated by patients.^44^ Providers implementing care in homeless shelters recommend a short course of HCV therapy for this group due to residents' transient lifestyles.^17^

## 4 DISCUSSION

This systematic review has identified a number of interacting barriers and facilitators to HCV testing and treatment among people who experience homelessness, especially in hospital settings. Some mirror those identified for the general population,^49^ others are unique to this group. The barriers and facilitators identified can guide the development of effective programmes tailored to their complex health needs.^18^ Some of the barriers identified may be difficult to address or take time to modify, for example the lived experience of homelessness and associated morbidities, or workforce constraints. Others such as lack of HCV awareness and limited advocacy are more amenable to change.^17^

In this review, we found access to HCV care for people who experience homelessness to be influenced by an interplay of individual and system characteristics. Although some barriers reflected those identified for the general population,^49^ the lived experience of homelessness, marked by precarious living conditions, disproportionately limits the ability of people to seek and receive HCV treatment. This exacerbates the barriers related to population characteristics. For example, while long wait times for appointments are barriers to HCV screening for all, the transiency of a homeless lifestyle renders it harder to remember and attend appointments. Similarly, while stigma and negative attitudes towards HCV persist for everyone, the stigmatizing perceptions attached to homelessness further impede the ability of people to seek care.

Ensuring equity of access will require efforts from mainstream healthcare services to adapt to the multiple and complex needs of this vulnerable group. This warrants flexible, targeted and integrated care models to enhance engagement with HCV care and optimize treatment outcomes.^17^, ^50^ Since homelessness and unstable housing are associated with increased risk of HCV infection among people who inject drugs,^51^ the importance of advocating for meeting basic human needs as a means of optimizing their care, should not be underestimated.

Consistent with studies in other high‐risk populations,^47^, ^52^ peer education has been suggested as a key component of any interventions to address knowledge gaps and improve engagement among people experiencing homelessness. In prison settings, prisoners who had completed HCV treatment were perceived as being in a good position to understand the treatment process and its impact.^53^ This highlights the importance of peer advocates having experience of HCV care in establishing rapport. Understanding by healthcare staff of the role of peer advocates and flexibility around rules of acceptable involvement by them can facilitate the attendance of clients at appointments.^48^ Peer advocates can also make the work of healthcare staff more efficient by saving time in explaining treatment details.^48^ Further research on the optimal engagement of peer support is needed, including using people with lived experience of homelessness and HCV to inform communication strategies. In addition, how peer supporters themselves can be supported after disclosing their own trauma and experiencing similar adversities to others, should be explored further.

Despite the irrefutable value of peer advocate involvement, this systematic review has identified a clear lack of involvement of people with lived experience in decisions about treatment planning, access and provision, or in research. Devaluation of such knowledge as compared to that of academics, practitioners and policy makers, leads to a lack of understanding of how inequalities operate and how these might be addressed.^54^ As highlighted in the ‘Homeless and inclusion health standards for commissioners and service providers’,^55^ involvement of ‘experts by experience’ in planning and delivery can ensure person‐centred care through services that respond to the needs of the particular patient group, and ensure the acceptability, sustainability and effectiveness of services.

A lack of awareness both of the disease and of advances in HCV treatment post interferon emerged as a dominant theme in this review. An earlier scoping review that focused on the general population also identified knowledge gaps among patients of all levels of risk,^49^ while enhanced patient awareness emerged as a key facilitator of HCV testing among homeless populations.^17^, ^18^ Notably, although lack of health insurance has been reported as a barrier to care for the general population,^49^ our review found that cost of DAA treatment or insurance coverage was not always perceived as a significant barrier,^18^ implying a more complex relationship between costs and treatment access/utilization.

Our findings clearly support a need for educational programmes, covering risk factors, and testing and treatment regimes, including advances in DAA treatment. These programmes should ideally include education on all blood‐borne viruses and target support staff in shelters and drug support services, as well as people experiencing homelessness. Having greater knowledge is associated with increased willingness to engage,^56^ with the potential to improve rates of DAA treatment, reduce transmission and improve health outcomes in people experiencing homelessness.

### 4.1 Strengths and limitations

This review adopted a robust and systematic approach to select studies and identify the barriers and facilitators to HCV screening and treatment for people with lived experience of homelessness. As well as making practical and research recommendations to optimise services, our review could inform relevant public health actions to enhance healthcare equity.

The use of Penchansky and Thomas's modified access model enabled a systematic and rigorous analysis of data, thus enhancing the replicability of our findings.^38^ However, some subthemes traversed multiple themes making it difficult to compartmentalize them. For example, although the cost was discussed in reference to affordability, the knowledge that treatment was free of charge could also fit under the awareness theme. This brings to light the complex and intersecting factors affecting HCV care in this population.

The information provided for each included study, can enable readers to assess the applicability of findings to other contexts, although transferability may still be limited, for example, to ethnic minorities and depend on a country's healthcare system. For example, lack of insurance coverage is more applicable to the US setting since HCV treatment is provided free of charge in the United Kingdom under the National Health Service. Appointment timings were commonly reported as barriers in the United Kingdom, while strict eligibility criteria were more applicable to the United States.

Although an extensive and systematic approach to searching was adopted, the possibility of missing relevant studies cannot be excluded. Bringing qualitative and quantitative data together into a single synthesis remains challenging due to the methodological diversity within and between qualitative studies.^35^ However, it is increasingly recognized that such methods are required to meet the needs of policy makers and practitioners whose decision‐making needs to benefit from the range of evidence available.^57^, ^58^

The critical appraisal results were not used to weigh the review findings, which raises the possibility of introducing bias in the results. Currently, there is no consensus on the use of critical appraisal in interpreting qualitative findings.^59^ The fact that in two studies there was a mixed population (of people injecting drugs and those experiencing homelessness) is also a potential source of bias.

### 4.2 Future research

Evidence on the effectiveness of models of HCV care for people experiencing homelessness is lacking. Future studies could review models of the effectiveness of HCV care among people experiencing homelessness and explore the factors that influence the integration of HCV care with other health and social care services. Also, considering the various types of homelessness, research into access to HCV care among people sleeping rough may be warranted.

The participants in the included studies were predominantly male and White/Caucasian. Yet females are reported to experience stigma associated with injecting drug use and HCV more deeply than males.^60^ Although the under‐representation of females in studies may be due partly to their representation in the population of people injecting drugs,^40^ analysis into gendered issues affecting HCV treatment is very limited. Researching gender‐specific needs could enable the development of tailored interventions that meet diverse characteristics and contexts. Furthermore, considering the increase in homelessness among Black, Asian and minority ethnic groups and recent studies showing a lower likelihood of DAA treatment initiation among minorities,^61^, ^62^ exploring the specific needs of this group is warranted.

## 5 CONCLUSIONS

People experiencing homelessness face multiple and interacting barriers in accessing and completing HCV treatment. Some of these are readily amenable to change, others are more difficult to modify. There is an ethical responsibility for making adjustments to minimize barriers in all settings. Outreach, flexibility and culturally informed and tailored approaches are key to the success of interventions and care pathways aiming to improve engagement and completion of HCV care. Research is warranted into successful models to promote screening, diagnosis, uptake and treatment for this group. Involvement of people with lived experience of homelessness in the development and implementation of services is an important way to ensure that their needs are met.

## CONFLICT OF INTERESTS

The authors declare that there are no conflict of interests.

## ETHICS STATEMENT

Ethical and patient consent statements are not applicable for this article.

## AUTHOR CONTRIBUTIONS

Martha Paisi made substantial contributions to study conception and design, and acquisition, analysis and interpretation of data; drafted the manuscript and revised it critically for important intellectual content; gave final approval of the version to be published; agreed to be accountable for all aspects of the work in ensuring that questions related to the accuracy or integrity of any part of the work are appropriately investigated and resolved. Neeltje Crombag made substantial contributions to study conception and design, and acquisition, analysis and interpretation of data; revised the manuscript critically for important intellectual content; gave final approval of the version to be published; agreed to be accountable for all aspects of the work in ensuring that questions related to the accuracy or integrity of any part of the work are appropriately investigated and resolved. Lorna Burns made substantial contributions to study conception and design, and acquisition of data; revised the manuscript critically for important intellectual content; gave final approval of the version to be published; agreed to be accountable for all aspects of the work in ensuring that questions related to the accuracy or integrity of any part of the work are appropriately investigated and resolved. Annick Bogaerts made substantial contributions to study conception and design; revised the manuscript critically for important intellectual content; gave final approval of the version to be published; agreed to be accountable for all aspects of the work in ensuring that questions related to the accuracy or integrity of any part of the work are appropriately investigated and resolved. Lyndsey Withers made substantial contributions to study conception and design, and interpretation of data; revised the manuscript critically for important intellectual content; gave final approval of the version to be published; agreed to be accountable for all aspects of the work in ensuring that questions related to the accuracy or integrity of any part of the work are appropriately investigated and resolved. Laura Bates made substantial contributions to study conception and design, and interpretation of data; revised the manuscript critically for important intellectual content; gave final approval of the version to be published; agreed to be accountable for all aspects of the work in ensuring that questions related to the accuracy or integrity of any part of the work are appropriately investigated and resolved. Daniel Crowley made substantial contributions to study conception and design, and interpretation of data; critically revised the manuscript critically for important intellectual content; gave final approval of the version to be published; agreed to be accountable for all aspects of the work in ensuring that questions related to the accuracy or integrity of any part of the work are appropriately investigated and resolved. Robert Witton made substantial contributions to study conception and design; revised the manuscript critically for important intellectual content; gave final approval of the version to be published; agreed to be accountable for all aspects of the work in ensuring that questions related to the accuracy or integrity of any part of the work are appropriately investigated and resolved. Jill Shawe made substantial contributions to study conception and design, and analysis and interpretation of data; revised the manuscript critically for important intellectual content; gave final approval of the version to be published; agreed to be accountable for all aspects of the work in ensuring that questions related to the accuracy or integrity of any part of the work are appropriately investigated and resolved.

## Supporting information

Supporting information.

Supporting information.

Supporting information.

Supporting information.

## Data Availability

All data generated or analysed during this study are included in this published article [and its supplementary information files].
