# Supplementary material for: Barriers and facilitators to hepatitis C screening and treatment for people with lived experience of homelessness: A mixed‐methods systematic review
Source: Health Expect. 2021 Dec 3;25(1):48–60. doi: 10.1111/hex.13400 (PMC8849376; doi:10.1111/hex.13400)
Supplement: Supplementary file 1 — Supporting information. [file HEX-25-48-s002.docx]

**Supplementary File 1. Search strategies**

| **Embase** <1974 to 2020 December 02> | |  |  |
| --- | --- | --- | --- |
| Search history sorted by search number ascending | |  |  |
| # | Searches | Results | Type |
|  |  |  |  |
| 1 | exp homeless person/ | 2339 | Advanced |
| 2 | homelessness/ | 11459 | Advanced |
| 3 | homeless*.ab,kw,ti. | 13646 | Advanced |
| 4 | houseless*.ab,kw,ti. | 9 | Advanced |
| 5 | (rough adj3 sleep*).ab,kw,ti. | 93 | Advanced |
| 6 | (street adj1 (person or people or youth)).ab,kw,ti. | 363 | Advanced |
| 7 | halfway house/ | 1016 | Advanced |
| 8 | hostel*.ab,kw,ti. | 1144 | Advanced |
| 9 | night shelter*.ab,kw,ti. | 46 | Advanced |
| 10 | winter shelter*.ab,kw,ti. | 11 | Advanced |
| 11 | (seek* adj1 shelter).ab,kw,ti. | 154 | Advanced |
| 12 | emergency accommodation.ab,kw,ti. | 24 | Advanced |
| 13 | temporary accommodation.ab,kw,ti. | 71 | Advanced |
| 14 | supported accommodation.ab,kw,ti. | 185 | Advanced |
| 15 | supported housing.ab,kw,ti. | 409 | Advanced |
| 16 | (vulnerab* adj1 hous*).ab,kw,ti. | 269 | Advanced |
| 17 | (precarious* adj1 hous*).ab,kw,ti. | 93 | Advanced |
| 18 | unstable housing.ab,kw,ti. | 539 | Advanced |
| 19 | underhous*.ab,kw,ti. | 11 | Advanced |
| 20 | roofless*.ab,kw,ti. | 17 | Advanced |
| 21 | no fixed abode.ab,kw,ti. | 59 | Advanced |
| 22 | no fixed address.ab,kw,ti. | 35 | Advanced |
| 23 | supported lodging.ab,kw,ti. | 1 | Advanced |
| 24 | ((sofa or couch) adj1 surf*).ab,kw,ti. | 37 | Advanced |
| 25 | (bed adj2 breakfast).ab,kw,ti. | 35 | Advanced |
| 26 | (liv* adj3 squat*).ab,kw,ti. | 50 | Advanced |
| 27 | squatter*.ab,kw,ti. | 332 | Advanced |
| 28 | runaway*.ab,kw,ti. | 1717 | Advanced |
| 29 | (risk adj3 evict*).ab,kw,ti. | 24 | Advanced |
| 30 | soup kitchen*.ab,kw,ti. | 111 | Advanced |
| 31 | or/1-30 | 21753 | Advanced |
| 32 | exp Hepatitis C/ | 118788 | Advanced |
| 33 | "Hepatitis C".ab,kw,ti. | 117646 | Advanced |
| 34 | "Hep C".ab,kw,ti. | 392 | Advanced |
| 35 | "HCV".ab,kw,ti. | 100349 | Advanced |
| 36 | 32 or 33 or 34 or 35 | 168770 | Advanced |
| 37 | 31 and 36 | 856 | Advanced |
| 38 | (conference or conference abstract or conference paper or "conference review" or editorial or letter or note).pt. | 7366722 | Advanced |
| 39 | 37 not 38 | 490 | Advanced |

| **Ovid MEDLINE**(R) and Epub Ahead of Print, In-Process & Other Non-Indexed Citations, Daily and Versions(R) <1946 to December 02, 2020> | | | |
| --- | --- | --- | --- |
| Search history sorted by search number ascending | |  |  |
| # | Searches | Results | Type |
|  |  |  |  |
| 1 | exp homeless person/ | 9190 | Advanced |
| 2 | homelessness/ | 7982 | Advanced |
| 3 | homeless*.ab,kf,ti. | 11138 | Advanced |
| 4 | houseless*.ab,kf,ti. | 8 | Advanced |
| 5 | (rough adj3 sleep*).ab,kf,ti. | 67 | Advanced |
| 6 | (street adj1 (person or people or youth)).ab,kf,ti. | 295 | Advanced |
| 7 | halfway house/ | 1060 | Advanced |
| 8 | hostel*.ab,kf,ti. | 836 | Advanced |
| 9 | night shelter*.ab,kf,ti. | 39 | Advanced |
| 10 | winter shelter*.ab,kf,ti. | 7 | Advanced |
| 11 | (seek* adj1 shelter).ab,kf,ti. | 143 | Advanced |
| 12 | emergency accommodation.ab,kf,ti. | 14 | Advanced |
| 13 | temporary accommodation.ab,kf,ti. | 56 | Advanced |
| 14 | supported accommodation.ab,kf,ti. | 122 | Advanced |
| 15 | supported housing.ab,kf,ti. | 333 | Advanced |
| 16 | (vulnerab* adj1 hous*).ab,kf,ti. | 243 | Advanced |
| 17 | (precarious* adj1 hous*).ab,kf,ti. | 79 | Advanced |
| 18 | underhous*.ab,kf,ti. | 9 | Advanced |
| 19 | unstable housing.ab,kf,ti. | 349 | Advanced |
| 20 | roofless*.ab,kf,ti. | 16 | Advanced |
| 21 | no fixed abode.ab,kf,ti. | 44 | Advanced |
| 22 | no fixed address.ab,kf,ti. | 27 | Advanced |
| 23 | (risk adj3 evict*).ab,kf,ti. | 22 | Advanced |
| 24 | supported lodging.ab,kf,ti. | 1 | Advanced |
| 25 | ((sofa or couch) adj1 surf*).ab,kf,ti. | 18 | Advanced |
| 26 | (bed adj2 breakfast).ab,kf,ti. | 26 | Advanced |
| 27 | (liv* adj3 squat*).ab,kf,ti. | 47 | Advanced |
| 28 | squatter*.ab,kf,ti. | 365 | Advanced |
| 29 | runaway*.ab,kf,ti. | 1677 | Advanced |
| 30 | soup kitchen*.ab,kf,ti. | 94 | Advanced |
| 31 | or/1-30 | 18066 | Advanced |
| 32 | exp Hepatitis C/ | 64808 | Advanced |
| 33 | "Hepatitis C".ab,kf,ti. | 78789 | Advanced |
| 34 | "Hep C".ab,kf,ti. | 65 | Advanced |
| 35 | "HCV".ab,kf,ti. | 59270 | Advanced |
| 36 | 32 or 33 or 34 or 35 | 98208 | Advanced |
| 37 | 31 and 36 | 416 | Advanced |
| 38 | (comment or congress or editorial or letter).pt. | 1971897 | Advanced |
| 39 | 37 not 38 | 405 | Advanced |

**CINAHL and SocIndex via EBSCOhost**

|  |  |  |
| --- | --- | --- |
| S1 | ( (SU "Homeless Persons") OR (SU "Homelessness") ) OR TI homeless* OR TI houseless* OR TI rough N3 sleep* OR TI ( street N1 (person* or people or youth*) ) OR TI "halfway house" OR TI hostel* OR TI ( (night OR winter) N3 shelter* ) OR TI "emergency accommodation" OR TI "supported accommodation" OR TI "temporary accommodation" OR TI "supported housing" OR TI "supported lodging" OR TI vulnerab* N1 hous* OR TI precarious* N1 hous* OR TI underhous* OR TI "unstable housing" OR TI roofless* OR TI "no fixed abode" OR TI "no fixed address" OR TI risk N3 evict* OR TI ((sofa OR couch) N3 surf*) OR TI bed N3 breakfast OR TI ( (live OR living) N3 squat* ) OR TI squatter* OR TI runaway* OR TI "soup kitchen* OR AB homeless* OR AB houseless* OR AB rough N3 sleep* OR AB ( street N1 (person* or people or youth*) ) OR AB "halfway house" OR AB hostel* OR AB ( (night OR winter) N3 shelter* ) OR AB "emergency accommodation" OR AB "supported accommodation" OR AB "temporary accommodation" OR AB "supported housing" OR AB "supported lodging" OR AB vulnerab* N1 hous* OR AB precarious* N1 hous* OR AB underhous* OR AB "unstable housing" OR AB roofless* OR AB "no fixed abode" OR AB "no fixed address" OR AB risk N3 evict* OR AB ((sofa OR couch) N3 surf*) OR AB bed N3 breakfast OR AB ( (live OR living) N3 squat* ) OR AB squatter* OR AB runaway* OR AB "soup kitchen* | 17,889 |
| S2 | SU "Hepatitis C" OR TI ( "Hepatitis C" or HCV or "Hep C" ) OR AB ( "Hepatitis C" or HCV or "Hep C" ) | 21,113 |
| S3 | S1 AND S2 | 165 |

| CINAHL 145 |
| --- |
| SocIndex 20 |
| 153 imported (Ebsco removed duplicates) |
