# Supplementary material for: Barriers and facilitators to hepatitis C screening and treatment for people with lived experience of homelessness: A mixed‐methods systematic review
Source: Health Expect. 2021 Dec 3;25(1):48–60. doi: 10.1111/hex.13400 (PMC8849376; doi:10.1111/hex.13400)
Supplement: Supplementary file 2 — Supporting information. [file HEX-25-48-s001.docx]

| **Supplementary File 2.** Table of characteristics | |  | |  | |  |  |  |  |  |  |  |  |
| --- | --- | --- | --- | --- | --- | --- | --- | --- | --- | --- | --- | --- | --- |
| **Author, year** | **Country** | | **Study design** | | **Setting** | | **Type of homelessness** | **Sampling** | **Data collection method (s)** | **Participant characteristics** | **Other demographic characteristics** | **Barriers** | **Facilitators** |
| **Peer-reviewed literature** |  | |  | |  | |  |  |  |  |  |  |  |
| Beiser et al., 2017 | USA | | Cross-sectional | | Four clinic site locations within Boston Health Care for the Homeless Programme (a freestanding outpatient clinic, a medical respite facility, a clinic located in a day shelter, and a clinic within an emergency shelter) | | HCV-infected individuals experiencing homelessness seeking medical, behavioural or case management services | Convenience | A unique 32- item self- administered anonymous questionnaire was developed on the basis of prior questionnaires, investigator- directed interests, and patient focus groups. | N= 240 patients with HCV Most respondents (43%) were aged between 45-54 years 80.1% identified as male; 49.2% were White, 24.6% Black or African American, 13.3% other etc. | Time since HCV diagnosis (N=225): ≤1 year: 12%; 1-5 years: 19.1%; 5-10 years: 25.8%; 10–20 years: 29.3%; >years: 13.8% Reported use of any drug in the past month: 41.2% Injected drugs in the past month: 19.6%  Housing: 44.2% shelter, 23.8% housed, 17.9% sleeping rough, 4.6% staying with family or friends, not on lease. |  | Acceptability |
| Fokuo et al., 2020 | USA | | Qualitative | | 4 large homeless shelters (serving 300-400 individuals per day) located in urban areas. The shelters provided a variety of services to their clients and limited HCV testing and linkage to HCV services. | | Marginally housed and homeless individuals. | Purposive-through shelter directors | Focus groups were conducted by experienced facilitators using a semistructured interview guide. | N=27 participants 16 were practice providers, 7 were social service outreach workers and 4 were shelter staff |  | Awareness, Acceptability, Accomodation, Affordability. | Accomodation, Awareness, Affordability,  Accessibility,  Availability. |

| **Author, year** | **Country** | **Study design** | **Setting** | **Type of homelessness** | **Sampling** | **Data collection method (s)** | **Participant characteristics** | **Other demographic characteristics** | **Barriers** | **Facilitators** |
| --- | --- | --- | --- | --- | --- | --- | --- | --- | --- | --- |
| Lambert et al.,2019 | Ireland | Qualitative | Safetynet Primary Care services (a network of general practitioners and nurses providing primary care to homeless and other marginalised groups). | Individuals using homeless services and hostels previously testing HCV positive in Phase 1 of 'HepCheck Dublin' (i.e. known positive) | Targeted sampling, a non-probability sampling framework | An in depth questionnaire comprising closed and open-ended questions | N=48 participants 69% were male | 78% were currently living in a hostel. The remaining were ‘sofa surfing’, sleeping rough or staying with friends. 85% were homeless for longer than 12 months. Average period of homelessness 6.2 years (range 2 months - 20 years); 69% reported use of drugs in the past 30 days, with 45% ever sharing needles and 73% currently attending a drug treatment centre.HCV infection status: 77% reported they were unaware, 9% reported that they had cleared the infection / attained sustained viral response (SVR),and 6% had ‘active’ infection. Regarding engagement with follow-up, among the 63% who had been previously referred to specialist care, 60% had attended at least one appointment. 50% of those who had attended specialist care previously had stopped attending. | Accomodation, Acceptability |  |
| MacLellan et al., 2017 | UK | Qualitative | Embedded withing the HALT: Hepatitis study, a RCT of a peer intervention for improving patient engagement with ational Health Services across London. | The client group were located among a hepatitis C-positive cohort of people who have a history of injecting drug use and homelessness. | Purposive | A single narrative interview conducted by the research nurse experienced in qualitative interviewing, on neutral premises with each of the five Pas. | N=5 PAs Aged on average 48 years All participants were males | Length of experience in the role as peer advocate: 1-3 years. All had experienced homelessness, substance misuse and mental health challenges. There was also personal experience of hepatitis C infection within the sample. |  | Accomodation |
| Masson et al., 2020 | USA | Qualitative | A large homeless shelter which provides services to over 300 people per day. The shelter provided supportive housing, meals and a variety of services, and limited HCV testing and linkage to HCV services. | Homeless shelter clients | Purposive | 2 sepate focus groups for men and women that followed a semi-structured interview format with open-ended questions. The facilitator used probes as needed to elicit more responses or expand ideas expressed by participants. | N=20 Age range: 26-69 years 10 females and 10 males Participants were predominantly racial/ethnic minorities (n = 15), while 5 identified as White | N=8 had history of injection drug use N=7 had been continuously living on the streets or in shelters without a place to stay for a year or longer. Among the 4 participants who disclosed their HCV status, 1 participant had chronic HCV infection but had not been treated, 1 had tested HCV antibody positive and did not have active infection, and 2 others who had chronic HCV infection had. | Awareness, Acceptability, Accomodation, Affordability | Awareness, Acceptability, Affordability |

| **Author, year** | **Country** | **Study design** | **Setting** | **Type of homelessness** | **Sampling** | **Data collection method (s)** | **Participant characteristics** | **Other demographic characteristics** | **Barriers** | **Facilitators** |
| --- | --- | --- | --- | --- | --- | --- | --- | --- | --- | --- |
| McGonigle et al., 2018 | USA | Cross-sectional | 2 large homeless shelters (and three residential substance abuse treatment centres)- however, they used data from the former) | Individuals accessing homeless shelters | Purposive | Retrospective chart review & qualitative free-form response. | N=509 participants were included in statistical analysis Average age: 42.7 years (range 19-79) 91.2% were male and 8.8% female; 38.1% identified as White, while 54.8% was Black. |  | Affordability, Accomodation, Accesibility, Acceptability |  |
| Stagg et al., 2019 | UK | Randomised non-blinded controlled trial | Outreach services for problematic drug use and homelessness for point of care HCV, HBV, and HIV testing. | Participants how were approached at outreach services for problematic drug use and homelessness and testing positive for HVC or HBV. | Not reported | The primary outcome of interest was successful achievement of an appropriate clinical endpoint, defined as engagement with clinical hepatitis services i.e. three engagements within 6 months of the first booked clinical appointment. | N= 364 consented to point-of -care. N=101 were enrolled with a chronic HCV infection by PCR. Of the 101 participants: 80.2% were males and 19.8% were females Majority were in the ages of 36-45 years (41.6%) and 46-55 years (34.7%) 69.3% were from White other ethnic background. | Of the 101: 78.2% were current users of ellicit drugs; 31.7% were currently injecting. 53.5% were 'known positives' at the time of recruitment. |  | Accomodation |
| Thompson et al., 2005 | USA | Qualitative | Research in Access to Care for the Homeless (REACH) cohort- single room occupancy hotels, homeless shelters and free lunch programs. | Homeless and marginally housed individuals. | Systematic | Face-to-face un-structured interviews. | N=52 primary healthcare providers of 133 patients who tested positive for HCV. 42%% were males and 58% were females. | 48.1% providers cared for one study patient, 44.2% cared for between two and five patients and 7.7% cared for 6 or more study patients. Median current patient panel: 700. The training included: general internal medicine (44%), infectious disease subspeciality (8%), family practice (8%), and nurse practitioner/physician’s assistant (21%). 37% practised in community clinics, 35% in hospital-based clinics, 13% in university-based clinics (13%), and 12% in private offices. | Accomodation |  |

| **Author, year** | **Country** | **Study design** | **Setting** | **Type of homelessness** | **Sampling** | **Data collection method (s)** | **Participant characteristics** | **Other demographic characteristics** | **Barriers** | **Facilitators** |
| --- | --- | --- | --- | --- | --- | --- | --- | --- | --- | --- |
| Williams et al., 2019 | USA | Qualitative | 2 healthcare for the homeless clinic settings. Both were multi-disciplinary primary care clinics that employ a patient -centred medical home model. | Non-cirrhotic patients from two groups-one receiving opioid agonist therapy (OAR), and another gorup frequentling a needle and syringe excahnge program (NSP)-majority of which had experienced homelessness. | Purposive | In-depth interviews with a subset of the participants of a pilot clinical trial testinf the effective delivery of DAA treatment to people ho inject drugs. | N=27 Mean age: 44 years 66.7% were males and 33.3% females Majority were Caucasian (n=26), with one participant identifying as American Indian and one as mixed Caucasian- Asian/Pacific-Islander | 70.4% had attended high school or less and 29.6% a BA/Trade school. Many participants (46.1%, n=12) were either homeless or in transitional housing. 51.9% reported the time of last drug use was within the past week at baseline. For 18.5% this was in the past 12 months and for 29.6% in over a year. |  | Acceptability |
| **Grey Literature** |  |  |  |  |  |  |  |  |  |  |
| HCV Action, 2018 | UK | Qualitative | An outreach hepatitis C treatment clinic established within a primary Care facility (the latter designed to provide healthcare services to patients who are homeless, at risk of becoming homeless and/or have extreme difficulty engaging with mainstream services). | Homeless patients infected with HCV. | Purposive | Interviews with patients. | N=6 patients at different stages of assessment/treatment, ranging from being at the beginning of the assessment process to having successfully completed treatment |  | Accessibility | Acceptability, Accessibility, Accomodation,  Availability. |
| London Joint Working Group, 2020 | UK | Qualitative | Outreach Hepatitis C testing and treatment interventions-Local Authority provided temporary accomodation to rough sleepers-as part of the 'Everyone In' policy. | Individuals living in hostels (accommodation specifically for homeless clients including supported housing schemes) and hotels where people were being temporarily accommodated. Other venues were day facilities, home visits and street outreach. | Purposive | Interviews with those involved in the planning and delivering of the interventions. | N=6 from The Hepatitis C Trust (N=4), Local Authoriry (N=1) and University College London Hospitals NHS Foundation Trust (N=1) |  | Awareness | Awareness, Accomodation,  Accessibility. |

| **Author, year** | **Country** | **Study design** | **Setting** | **Type of homelessness** | **Sampling** | **Data collection method (s)** | **Participant characteristics** | **Other demographic characteristics** | **Barriers** | **Facilitators** |
| --- | --- | --- | --- | --- | --- | --- | --- | --- | --- | --- |
| WHO, 2012 | UK | Qualitative | Site A-An established community-based partnership offering HCV treatment delivery and support for PWID and Site B- a nascent partnerhsip about to pilot a HCV outreach programme. | People who inject drugs in drug and alcohol settings, the majority of which were homeless or in unstable housing. | Purposive | In depth interviews with service users and service providers | N=49 N= 35 service users Mean age: 45.3 years (range 26-60) 17% were females 51.4% identified as White British, 40% as White other, 6% as Black British, 3% as mixed race British N=14 service providers; Consultant hepatologist (N=2); Consultant psychiatrisy (N=1); D&A nurse key worker (team leader) (N=2); BBV Nurse (N=4); Hospital-based hepatitis nurse (N=2); Administrator (N=1); Pharmaceutical company representative (N=1) | Service users: 68.6% reported living in independent or supported housing, 11.4% in hostel, 14.3% in unstable housing, 5.7% sleeping rough. Three of participants were employed full time, four were receiving Job Seekers Allowance of Income Support, 27 were receiving incapacity benefit, and one participant was a pensioner. 8 people reported currently injecting drugs, while 27 had stopped injecting. Thirty-two individuals were currently engaged in opiate substitution therapy (OST). The remaining three were not currently engaged in OST, but had been in the past. 29 individuals had no prior experience of HCV treatmen prior to that service. Five were contemplating whether or not to have treatment and six were waiting to start treatment. | Acceptability, Accomodation, Affordability,  Availability. | Awareness, Acceptability, Accomodation, Affordability,  Accessibility,  Availability. |
