# Supplementary material for: Barriers and facilitators to hepatitis C screening and treatment for people with lived experience of homelessness: A mixed‐methods systematic review
Source: Health Expect. 2021 Dec 3;25(1):48–60. doi: 10.1111/hex.13400 (PMC8849376; doi:10.1111/hex.13400)
Supplement: Supplementary file 3 — Supporting information. [file HEX-25-48-s003.docx]

**Supplementary File 3.**

Critical appraisal

| **CASP for qualitative studies** | **Was there a clear statement of the aims of the research?** | **Is a qualitative methodology appropriate?** | **Was the research design appropriate to address the aims of the research?** | **Was the recruitment strategy appropriate to the aims of the research?** | **Were the data collected in a way that addressed the research issue?** | **Has the relationship between researcher and participants been adequately considered?** | **Have ethical issues been taken into consideration?** | **Was the data analysis sufficiently rigorous?** | **Is there a clear statement of findings?** |
| --- | --- | --- | --- | --- | --- | --- | --- | --- | --- |
| **Peer-reviewed literature** | | | | | | | | | |
| **Fokuo et al., 2020** | Yes | Yes | Yes | Yes | Yes | Can't tell | Yes | Yes | Yes |
| **Lambert et al.,2019** | Yes | Yes | Can't tell | Yes | No | No | Yes | No | No |
| **MacLellan et al., 2017** | Yes | Yes | Yes | No | Yes | Yes | Yes | Yes | Yes |
| **Masson et al., 2020** | Yes | Yes | Yes | Can't tell | Yes | Yes | Yes | Yes | Yes |
| **Thompson et al., 2005** | Yes | Yes | No | Yes | Can't tell | No | Yes | No | No |
| **Williams et al., 2019** | Yes | Yes | Yes | Yes | Yes | Can't tell | Yes | Yes | Yes |
| **Grey Literature** | | | | | | | | | |
| **HCV Action, 2018** | Yes | Yes | Can't tell | Can't tell | Can't tell | No | Can't tell | No | Yes |
| **London Joint Working group, 2020** | No | Yes | No | Can't tell | Can't tell | Can't tell | No | No | No |
| **WHO, 2012** | Yes | Yes | Yes | Yes | Yes | Can't tell | Yes | Yes | Yes |

| **JBI for cross sectional studies** | **Were the criteria for inclusion in the sample clearly defined?** | **Were the study subjects and the setting described in detail?** | **Was the exposure measured in a valid and reliable way?** | **Were objective, standard criteria used for measurement of the condition?** | **Were confounding factors identified?** | **Were strategies to deal with confounding factors stated?** | **Were the outcomes measured in a valid and reliable way?** | **Was appropriate statistical analysis used?** |
| --- | --- | --- | --- | --- | --- | --- | --- | --- |
| **Peer-reviewed literature** | | | | | | | | |
| **Beiser et al., 2017** | No | Yes | No | No | No | No | No | Yes |
| **McGonigle et al., 2018** | No | Yes | Can't tell | No | No | No | No | No |

| **CASP for RCTs** | **Did the study address a clearly focused research question?** | **Was the assignment of participants to interventions randomised?** | **Were all participants who entered the study accounted for at its conclusion?** | **Were the participants ‘blind’ to intervention they were given?** | **Were the investigators ‘blind’ to the intervention they were giving to participants?** | **Were the people assessing/analysing outcome/s ‘blinded’?** | **Were the study groups similar at the start of the randomised controlled trial?** | **Apart from the experimental intervention, did each study group receive the same level of care (that is, were they treated equally)?** | **Were the effects of intervention reported comprehensively?** | **Was the precision of the estimate of the intervention or treatment effect reported?** | **Do the benefits of the experimental intervention outweigh the harms and costs?** |
| --- | --- | --- | --- | --- | --- | --- | --- | --- | --- | --- | --- |
| **Peer-reviewed literature** | |  |  |  |  |  |  |  |  |  |  |
| **Stagg et al., 2019** | Yes | Yes | Yes | No | No | No | Yes | Yes | Yes | Yes | Yes |
