# Supplementary material for: Barriers and facilitators to hepatitis C screening and treatment for people with lived experience of homelessness: A mixed‐methods systematic review
Source: Health Expect. 2021 Dec 3;25(1):48–60. doi: 10.1111/hex.13400 (PMC8849376; doi:10.1111/hex.13400)
Supplement: Supplementary file 4 — Supporting information. [file HEX-25-48-s004.docx]

**Supplementary File 4.** Illustrative quotations for each domain

| **Domain** |  | **Quotation and reference** |
| --- | --- | --- |
| **Awareness** | Barrier | “Is there a cure or treatment?” (homeless shelter client)^1^  “I feel like […] the narrative that is out there on the street is the battle days of interferon, everyone was told to just wait with their Hep C. They found out they were living with Hep C, and they (doctors) said, ‘well you know it’s never an emergency and just wait on it.” (key stakeholder)^2^ |
|  | Facilitator | “What motivated me to get tested is so it doesn’t spread.” (homeless shelter client).^1^  In particular, circulating information packs about BBVs, what was going to happen on the day and why it was important to get tested in the week before the event saved time on the day and encouraged uptake of the testing. (author)^3^ |
| **Acceptability** | Barrier | “They’re making you a guinea pig. I don’t trust [them]. Because this trust has been breached already.” (homeless shelter client).^1^  “Hep C, it’s much more of a stigma about injecting and being an injecting drug user and therefore, it’s dirty, you know, you’ve got it yourself, you’ve gone out and got it.” (service user)^4^  “We have people (saying) like oh ‘it’s ninety thousand dollars for treatment, aw I’ll never get that because I’m just this humble homeless person.’ They’ll just kind of (have) Stockholm syndrome that they (think they) are worthless people.” (key stakeholder)^2^ |
|  | Facilitator | Our findings highlight the need to address stigma in this vulnerable population through culturally informed treatment that is based on a trusting relationship between the patient, provider, and health care system (author).^1^  Indeed, every participant interviewed mentioned the familiarity of EAP and their relationship with staff as a reason for their positive view of the EAP hepatitis C clinic. (author)^5^    “[My concept of health] changed all throughout treatment. Like before I ate a lot of unhealthy food. I wouldn't really take care of my body as good. I also just made my first counselling appointment for my mental health, and I'm trying to get in for a psych eval, and so I think I changed a lot. I don't know why it's really strange it just happened to be the timing. I feel like okay now I'm getting my health going, and I think it motivated me to want to do more especially when you guys said that the first time my blood work came back” (person injecting drugs receiving treatment at a homeless clinic setting)^6^  Among respondents interested in treatment, the  most commonly identified motivating factors were “taking care of your health” (73.5%) and “your provider says you should get treated” (53.6%). (author)^7^  “Well they’re always their priorities but a lot of our patients, their life will always be some major thing going on like that. So that will not change for them, they’ll always be homeless or in a hostel or whatever. You can’t wait until they’re more stable, this may never happen, we’ve got to work with that...” (service provider)^4^ |
| **Accommodation** | Barrier | “They were pretty strict, because they’re so busy. Then that’s what they explained to me, that you can’t just come in, because I got to a point when I was coming in at different times and that, the right day but not at the exact time. I didn’t realise how booked up and busy they are and it’s one of the busiest departments I think in [hospital].” (service user)^4^  “Some people are stuck in addiction, that they’re not even caring about it [other medical conditions], they’re just looking towards the next fix or their next high.” (homeless shelter client) ^1^  “It’s a very chaotic world (for patients) and there’s competing priorities. I gotta eat; I got to find some shoes.” (key stakeholder)^2^  When asked about their HCV healthcare pathway, unstable accommodation was reported to be the most common barrier to attending specialist appointments and accessing treatment. (author)^8^  Depression (43.6%), drug use (33.0%) and alcohol consumption (19.1%) were frequent reasons to withhold therapy. (author)^9^ |
|  | Facilitator | “I’ve had drug and alcohol problems, mental health problems, so all of that helps me to empathise, you know... I can say ‘I know what you mean’ and mean it.” (peer advocate).^10^  Peer support with this group has increased engagement and supported many people into treatment. (author)^3^ |
| **Affordability** | Barrier | “The first thing that came through my mind was how was I going to afford this medication? Does Medicaid cover [the medication]?...If you don’t know what to ask for [the doctor], a lot of people will turn around and walk out the door.” (homeless shelter client)^1^  “I did a lot of communications with them (insurance companies) and the pharmacies about medications for hepatitis C and I think that was a big thing that took up a lot of our time. And clients didn’t want to sit for these interviews and questions.” (key stakeholder)^2^ |
|  | Facilitator | “People will get it [testing] as long as there is an incentive behind it so if there’s no incentive they won’t get it.” ( homeless shelter client).^1^  The potential availability of home and transport support for the treatment duration could act as an incentive for service users to consider treatment uptake, and may make the difference between treatment completion and non-completion for some. (author)^4^ |
| **Accessibility** | Barrier | “A doctor can do their job better if they know your past, obviously ... I think it’s important to keep seeing the same person. When you keep getting shoved from pillar to post all the time and you’re having to explain everything all over again, it’s much better to see the same person all the time and then they know where you’re up to and you haven’t got to keep saying it all.” (service user)^4^  “Some patients said that they would have had difficulty attending hospital appointments due to the geographical distance involved in travelling there. (author)^5^ |
|  | Facilitator | The geographical proximity of the clinic to patients was also identified as a positive aspect of the clinic”. (author)^5^ |
| **Availability** | Barrier | “So there is an anxiety around all that but there’s an anxiety about going into another environment, about waiting. They hate waiting because they’re always busy, they’re always – very often, their addiction plays a part in that. They’re also, I think it’s about – I mean I have anxiety going to my doctor or anybody but I think there’s an increased anxiety because they are socially less – more inept when it comes to dealing with professionals”. (service provider)^4^ |
|  | Facilitator | “Because in this population, they are getting moved around a lot, right? And so a shelter bed might be for a short period of time. It’s better to have a shorter course.” (key stakeholder)^2^ |

**References**

1. Masson CL, Fokuo JK, Anderson A, et al. Clients' perceptions of barriers and facilitators to implementing hepatitis C virus care in homeless shelters. *BMC Infect Dis.* 2020;20(1):386.

2. Fokuo JK, Masson CL, Anderson A, et al. Recommendations for Implementing Hepatitis C Virus Care in Homeless Shelters: The Stakeholder Perspective. *Hepatol Commun.* 2020;4(5):646-56.

3. London Joint Working Group on substance use and hepatitisC. *Hepatitis C testing and treatment interventions for the homeless population in London during the Covid-19 pandemic: Outcomes and learning.* London: London Joint Working Group on substance use and hepatitisC; 2020.

4. WHO. *Barriers and facilitators to hepatitis C treatment for people who inject drugs. A qualitative study.* Copenhagen: WHO; 2012.

5. HCVAction. *Edinburgh Access Practice outreach service. An example of treatment outreach for the homeless community.* Edinburgh: HCV Action; 2018.

6. Williams BE, Nelons D, Seaman A, et al. Life projects: the transformative potential of direct-acting antiviral treatment for hepatitis C among people who inject drugs. *Int J Drug Policy.* 2019;72:138-45.

7. Beiser M, Leon C, Gaeta JM. Needs Assessment of HCV-Infected Individuals Experiencing Homelessness and Implications. *J Health Care Poor Underserved.*2017;28(1):596-606.

8. Lambert JS, Murtagh R, Menezes D, et al. 'HepCheck Dublin': An intensified hepatitis C screening programme in a homeless population demonstrates the need for alternative models of care. *BMC Infect Dis.* 2019;19(1):128.

9. Thompson VV, Ragland KE, Hall CS, et al. Provider assessment of eligibility for hepatitis C treatment in HIV-infected homeless and marginally housed persons. *AIDS* 2005;19:S208-S14.

10. MacLellan J, Surey J, Abubakar I, et al. Using peer advocates to improve access to services among hard-to-reach populations with hepatitis C: A qualitative study of client and provider relationships. *Harm Reduct J.* 2017;14(1):76.
